# Understanding the Relative Contributions of Sensitive and Insensitive Parent Behaviors on Infant Vaccination Pain

**DOI:** 10.3390/children5060080

**Published:** 2018-06-18

**Authors:** Shaylea Badovinac, Hannah Gennis, Rebecca Pillai Riddell, Hartley Garfield, Saul Greenberg

**Affiliations:** 1 Department of Psychology, Faculty of Health, York University, 4700 Keele Street, Toronto, ON M3J 1P3, Canada; sdbadov@yorku.ca (S.B.); hgennis@yorku.ca (H.Ge.); 2 Department of Psychiatry Research, The Hospital for Sick Children, 555 University Avenue, Toronto, ON M5G 1X8, Canada; 3 Department of Psychiatry, University of Toronto, 27 King’s College Circle, Toronto, ON M5S 3H7, Canada; 4 Department of Pediatrics, University of Toronto, 27 King’s College Circle, Toronto, ON M5S 3H7, Canada; hartley.garfield@gmail.com (H.Ga.); saulped@yahoo.com (S.G.); 5 Department of Pediatrics, The Hospital for Sick Children, 555 University Avenue, Toronto, ON M5G 1X8, Canada

**Keywords:** parent sensitivity, parent insensitivity, vaccination, infant pain, pain management, parent, soothing

## Abstract

Parents play a critical role in supporting infants’ ability to manage strong emotions. Routine vaccinations provide an ideal context to observe the effect of parents’ behaviors on infants’ pain-related distress. Previous research in the vaccination context showed that parent sensitivity, operationalized by variables such as emotional availability and proximal soothing behaviors, is associated with infant pain-related distress behavior. However, the magnitudes of these relationships were smaller than expected given the established importance of parents in the development of distress regulation. In recent work, a reliable and valid measure to operationalize insensitive behaviors was developed. The objective of the current study was to examine the relative contribution of variables representing sensitive and insensitive behaviors to the prediction of infant pain-related distress behaviors during the reactivity and regulation phases of needle pain. Archival data was used to analyze a subsample of infants followed during their two-month, six-month, and 12-month vaccinations (*n* = 81). Results of regression analyses indicated that parent insensitive behaviors generally had the strongest relationships with pain outcomes across all ages, with a greater influence on regulation-phase pain-related distress behavior, rather than reactivity-phase pain-related distress behavior. Our findings support the utility of a measure of distress-promoting parent behaviors in a vaccination context, and highlight the potential value of this measure for clinicians and researchers.

## 1. Introduction

Emotion regulation, defined as one’s ability to select and enact appropriate strategies in the management of extreme emotions [1], underlies adaptive socioemotional development [2]. Difficulties with this skill, and particularly with the regulation of negative emotions, (i.e., distress regulation) were conceptualized as a transdiagnostic challenge underlying a significant proportion of mental health problems across the lifespan [3]. The foundations of distress regulation skills are laid during infancy and early childhood [4], with parents being major agents in this process [4,5]. Particularly during the first year of life [4], before children begin developing self-directed regulation strategies, parents and caregivers play a critical role in scaffolding this skill by modeling adaptive emotional responses to distress [5], and by providing external regulation strategies to support infants’ distress regulation [4,5]. Emotion regulation skills comprise one component of a broader repertoire of skills that support regulation from a distressing stimulus. Adaptive regulation involves physiological and behavioral components, in addition to cognitive-affective components. In young infants, basic forms of emotion regulation can be operationalized in terms of behavioral and physiological regulation.

Previous research used infant vaccinations as a paradigm to study infants’ developing pain-related distress regulation capacity. This paradigm has unique benefits as it offers a naturalistic, ethical, and universally standardized setting in which children’s pain-related distress regulation capacity can be observed in the context of parent behaviors. Infants’ behavior in response to a painful stimulus was conceptualized as involving two distinct, yet interrelated phases: the infant’s immediate behavioral response following the painful stimulus (i.e., pain-related distress reactivity), and the more distal or non-immediate behavioral response (i.e., pain-related distress regulation) [6,7]. Infant behavior during the reactivity phase is largely driven by biological and genetic factors, in addition to previous pain experiences, whereas behavior during the regulation phase is more closely linked to broader contextual factors, such as parent pain-management behaviors [7].

In light of the critical influence that parents have on infants’ pain-related distress regulation capacity, considerable efforts were dedicated to identifying the qualities of parents and parent–infant interactions that predict more optimal infant distress regulation within the vaccination context [6,8,9,10,11,12]. Much of the focus of these efforts was on discrete parent soothing behaviors, and resulted in mixed findings. Parent soothing behaviors can be differentiated into proximal soothing behaviors, which involve physical contact between caregiver and infant, and distal soothing behaviors, which do not involve physical contact (e.g., soothing sounds and verbal reassurance). While two studies identified significant relationships between proximal soothing behaviors, such as rocking and holding, and infant pain-related distress [6,8], other studies found that, after controlling for infant distress prior to and immediately following the vaccination needle, the contribution of proximal soothing behaviors to pain outcomes was minimal [10,12]. However, in line with previous theory [6,7], proximal soothing behaviors typically account for more of the variance in distress regulation, as opposed to distress reactivity [6,10].

An alternative line of research focused on the role of parent emotional availability within an infant vaccination context [13,14,15]. Grounded in attachment theory, the construct of emotional availability takes the dyadic nature of the parent–infant interaction into account, emphasizing the degree of emotional attunement between parent and infant [16]. Parental sensitivity, structuring, non-hostility, and non-intrusiveness are characteristics of parents with high emotional availability. Parental sensitivity describes the appropriateness of a parent’s verbal and non-verbal behaviors in the context of the child’s expressed needs and emotions. Structuring describes the extent to which the caregiver appropriately scaffolds and promotes the child’s autonomy. Non-hostility refers to the absence of concealed and covert forms of hostility (e.g., impatience, anger, and threatening or frightening actions or words). Non-intrusiveness describes the extent to which the caregiver refrains from over-stimulating the child or interfering with the child’s autonomy. Paralleling findings from the attachment literature, findings within the vaccination context suggest that parent emotional availability is associated with less infant pain-related distress during the regulation period [15], but this association isn’t reliably formed until the end of the first year of life [13,14], which is when parent–infant attachment bonds can be reliably measured [17].

Although considerable progress was made toward elucidating the parent behaviors that support an infant’s capacity to regulate distress, there are still significant gaps in our understanding of parents’ role in pain management. For example, previous research showed that, after controlling for infant pain reactivity, parent soothing behaviors ultimately account for less than 13% of the overall variance in outcomes [12]. This suggests that the aspects of parent behavior that are traditionally studied in a vaccination context may not capture the full extent of parents’ influence on infant pain outcomes. More recently, a different aspect of parent behavior was examined in an infant vaccination context [18]. Referred to as distress-promoting behaviors, these are behaviors that, while potentially well intentioned, have the paradoxical effect of exacerbating post-needle distress in infants during both the reactivity and regulation phases. When enacted by a parent in response to moderate to high infant distress, these behaviors are hypothesized to directly undermine the infant’s needs for contingent physical and emotional responding from the parent. This lack of attunement between the child’s needs and the parent’s response is seen as being reflective of insensitive parent behavior. In support of this premise, strong negative relationships were found between these insensitive or distress-promoting parent behaviors and parent emotional availability [18].

Although insensitive parent behaviors appear to be a key factor contributing to infant pain-related distress, an understanding of the influence of these behaviors in the context of other more optimal behaviors (e.g., emotional availability and proximal soothing) is needed. Among preschoolers [19] and older children [20,21], when parent distress-promoting and coping-promoting behaviors were assessed concurrently in a vaccination context, distress-promoting behaviors were consistently shown to have stronger relationships with pain-related distress. This suggests that, in the context of toddler and child vaccinations, instructing parents to avoid engaging in distress-promoting behavior may be more important than instructing parents to engage in coping-promoting behaviors [19]. Whether this pattern holds for infant pain-related distress is unclear. Although previous research involving infant samples seems to suggest strong relationships with distress-promoting parent behaviors [18], and significant but small relationships with coping-promoting (e.g., emotional availability [13,14,15] and proximal soothing [6,8,9,10]) behaviors, these behaviors were not examined concurrently, to our knowledge. 

The objective of the current study was to examine the relative contributions of optimal (i.e., emotional availability and proximal soothing; sensitive behaviors) and suboptimal (i.e., distress-promoting; insensitive behavior) parent behaviors in predicting infants’ pain-related distress during the first year of life, using archival data from a large longitudinal vaccination cohort (the Opportunities to Understand Childhood Hurt, OUCH cohort [13]). We had two primary research questions:

1. What are the relative contributions of parent emotional availability, proximal soothing, and distress-promoting behaviors to predicting infant pain behaviors during the reactivity phase during two-month, six-month, and 12-month vaccinations?
2. What are the relative contributions of parent emotional availability, proximal soothing, and distress-promoting behaviors to predicting infant pain behaviors during the regulation phase during two-month, six-month, and 12-month vaccinations?

Based on the previous research reviewed earlier, we hypothesized the following:

1. Across all ages, the model predicting infant pain behaviors during the regulation phase from parent behaviors would account for more variance than the model predicting infant pain during the reactivity phase.
2. Parent distress-promoting behaviors would account for more variance in infant pain behaviors during both the reactivity and regulation phases, across all ages.

## 2. Materials and Methods

### 2.1. Participants

Participants in the current study were part of the Opportunities to Understand Childhood Hurt (OUCH) cohort. Parent–infant dyads included in the current analyses were recruited from three pediatric clinics in Toronto, Canada, between October 2007 and May 2012. For the current study, a subsample of dyads that had parent and infant behavioral data on the five variables of interest at the two-month, six-month, and 12-month vaccination appointments were included. Due to missing data (i.e., incomplete or uncodable video footage), some dyads were missing from the regression analyses at each time point (*n* = 14 at two months, *n* = 7 at six months, and *n* = 2 at 12 months). No previously published or planned/submitted manuscripts from this cohort had hypotheses or analyses that overlapped with the current study.

Parent–infant dyads were eligible to participate if the infant was born full-term (≥37 weeks), had no suspected developmental delays, impairments, or chronic illnesses, and was never admitted to a neonatal intensive care unit, and if the parent was able to speak and read English. The original sample included 760 parent–infant dyads who were followed at routine vaccinations throughout the first year of life (for full sample demographics, see References [13,19]). 

For the current study sample (*n* = 81), the mean age of parents at recruitment (i.e., at two-month vaccinations) was 33.12 years old (SD = 5.30; range = 19–45). The majority of parents in this sample were married (84%), and had a university degree or higher (74.1%). Parents’ self-reported heritage culture was diverse; 37.2% self-reported as European, 15.4% as Asian, 11.5% as African/Middle Eastern, 10.3% as North American, 6.4% as Jewish, 2.6% as South/Latin American, 5.1% as mixed, and 11.5% as other. Across appointments, the average number of needles given to infants was 1.92 (SD = 0.40; range = 1–3). Table 1 presents additional demographic information for the dyads analyzed at each time point.

### 2.2. Procedure

Ethics approval was obtained from the affiliated university and the associated pediatric hospital (Research Ethics Board Certificate Number: 2007-203). Full details of the procedure were published elsewhere [13]. Thus, only a brief summary of the procedures relevant to the current study will be provided. Parents and healthy, typically developing infants in the current study were recruited during the infants’ two-month, four-month, or six-month well-baby vaccination appointments, and were followed for remaining vaccination appointments over the first year of life. After informed consent was obtained, dyads were videotaped during the appointment from the moment the dyad entered the examination room until up to five minutes after the vaccination. Parents were naturalistically observed, and researchers allowed parents to soothe their child in whatever way they chose. Lisi et al. [12] described the chosen soothing strategies utilized by parents in the OUCH cohort. Following each appointment, parents were mailed a copy of their infant’s vaccination video. Demographic information and video footage were collected at all three appointments, and trained coders coded parent proximal soothing behaviors, emotional availability, and distress-promoting behaviors, as well as infant pain-related distress, for each appointment. Coders for each measure were blind to the study hypotheses, and to parent and infant scores on other measures.

### 2.3. Apparatus

Two Canon HD Video Camcorders (HV20) were used to obtain video footage. One camera was fitted with a wide-angle lens, and mounted on a tripod to capture the entire interaction (i.e., parent and infant). This footage was used to code parent emotional availability, proximal soothing, and distress-promoting behaviors. The second camera was held by a research assistant, and was used to capture a close-up video of the infant. This footage was used to code infant pain-related distress.

### 2.4. Measures

#### 2.4.1. Demographic Questionnaire

Parents completed a demographic questionnaire that asked for basic background information (i.e., age, education, occupation, income, and self-reported heritage culture), as well as infant age, gender, and the parent’s relationship to the infant.

#### 2.4.2. Infant Pain Behaviors

Infant pain-related distress behaviors during the reactivity and regulation phases were measured using the Modified Behavioral Pain Scale (MBPS [22]). This scale uses three observable behavioral indices (facial expression, crying, and body movement) to approximate the amount of pain an infant is experiencing. The intensity of each behavior is rated by trained coders during 15-second epochs, based on video footage. Facial expression and body movement were each rated on a scale of 0–3, and crying was rated on a scale of 0–4. These scores were summed to generate a total score ranging from 0–10 for each 15-second epoch, with higher scores reflecting greater infant pain-related distress. Moderate-to-high concurrent and construct validity, as well as item-total and inter-rater reliability, were found when using this measure in an vaccination context [22]. 

MBPS was coded during three 15-second epochs, which occurred (1) immediately following the last vaccination needle, (2) at one minute post-needle, and (3) at two minutes post-needle. The first epoch was used to represent infant pain behaviors during the reactivity phase, with scores ranging from 0–10. The sum of the latter two epochs was used to represent infant pain behaviors during the regulation phase, with scores ranging from 0–20. Inter-rater reliability was high, with intra-class correlations ranging from 0.93–0.96.

#### 2.4.3. Parent Proximal Soothing Behavior

Parent proximal soothing behavior was measured using the Measure of Adult and Infant Soothing and Distress (MAISD [23]). The MAISD is a valid and reliable behavioral observation scale that was developed to evaluate the behavior of infants, parents, and health care professionals during painful pediatric medical procedures. For the purposes of the current study, only parent behaviors related to proximal soothing were included. This included two parent behaviors: physical comfort and rocking. Physical comfort was coded when the parent engaged in any physical (i.e., non-verbal) behavior in an attempt to comfort the infant. This included rubbing, massaging, patting, hugging, or kissing the infant. Rocking was coded when the parent swayed, rocked, or bounced the infant.

Each behavior was rated as present (1) or absent (0) during five-second epochs spanning the three minutes following the last vaccination needle. For each behavior, index scores ranging from 0 to 1 were calculated for each one-minute phase, by summing the number of five-second epochs during which the behavior was present, and by dividing the sum by the number of codable epochs during the one-minute period. Index scores for each behavior (i.e., physical comfort and rocking) during each phase (i.e., one, two, and three minutes post-needle) were summed for a total score ranging from 0–6, with higher scores reflecting a higher frequency of parent proximal soothing behavior. Intra-class correlations ranged from 0.86–0.92 for rocking, and from 0.72–0.88 for physical comfort.

#### 2.4.4. Parent Emotional Availability 

Parent emotional availability was measured using the Infancy/Early Childhood Version of the Emotional Availability Scale (EAS), Fourth Edition [24]. The EAS is a global clinical judgment of caregiving behavior through the examination of four parent subscales (sensitivity, structuring, non-instrusiveness, and non-hostility) which takes into account the infant’s responses to the parent’s behavior. It was validated in distressing non-pain [25] and pain [11,13,15] contexts.

Footage from the entire vaccination appointment was used to code EAS. Within each subscale, parents received a score ranging from 7–29. Scores from each of the four subscales were then combined to generate an overall emotional availability composite ranging from 28–116, with higher scores reflecting a more optimal emotional relationship between the parent and infant [25]. Intra-class correlations ranged from 0.83–0.94, suggesting good inter-rater reliability.

#### 2.4.5. Parent Distress-Promoting Behaviors

Parent distress-promoting behaviors were assessed using a checklist of observable distress-promoting behaviors with established validity and reliability in a vaccination context [18]. The checklist examines the presence of eight suboptimal or insensitive parent behaviors: (1) fathom wrong (i.e., making comments toward the distressed infant that either do not address, or discredit the infant’s distress, such as saying “It’s not so bad”), (2) face cover (i.e., covering the distressed infant’s face with any object such as a hand or blanket), (3) fashion first (i.e., dressing a distressed infant with no attempt to soothe the infant), (4) forceful (i.e., handling the infant roughly), (5) frustration (i.e., any facial expressions that reflect irritation with the infant’s distress, such as rolling eyes or sighing), (6) fearful (i.e., any parental facial expression that suggests they are scared or frightened), (7) flit away (i.e., any behavior or parental positioning that does not bring the infant close to the parent when the infant is distressed), and (8) flat face (i.e., a complete lack of emotional expression in response to the infant’s distress). Each behavior could only be coded when the infant was in moderate-to-high distress, with the exception of forceful, which could be coded at any time during the interaction. 

Each of the eight behaviors was rated as present (1) or absent (0) during a three-minute period following the last needle. This yielded a total score ranging from 0–8, with higher scores reflecting more distress-promoting parent behaviors. Intra-class correlations ranged from 0.73–0.98 across the three time points, reflecting good inter-rater reliability.

### 2.5. Statistical Analyses

To examine the relative contribution of parent proximal soothing, emotional availability, and distress-promoting behaviors to the prediction of infant pain behaviors during the reactivity and regulation phases at each infant age, multiple linear regression analyses using the “enter” (i.e., simultaneous) method were conducted. To screen for evidence that the assumptions of linearity and homoscedasticity were violated, scatter plots of predictors and outcomes, predictors and residuals, and residuals with predicted values of the outcome were examined. To screen for multicollinearity, intercorrelations between all predictor variables were conducted, and a conservative cut-off criterion of 0.7 was used to identify multicollinear variables. To examine the overall variance accounted for by each regression model, adjusted coefficients of determination (adjusted R^2^) were used. To examine the unique contribution of each predictor variable within each model, squared semi-partial correlations (*sr*^2^) were calculated. Within all presented results, *F* represents the outcome test statistic from the regression analysis and β represents the standardized regression coefficient. All analyses were conducted using the IBM SPSS Statistics software (Version 22.0.0.0).

## 3. Results

Descriptive statistics for all infant and parent variables are provided in Table 2. A total of six regression analyses were conducted in order to predict infant pain behaviors during the reactivity and regulation phases at two months, six months, and 12 months.

### 3.1. Research Question 1: Predicting Infant Pain Behaviors during the Reactivity Phase

To investigate the relationship between insensitive and sensitive parent variables, and infant immediate pain reactivity, multiple linear regressions were conducted using parent emotional availability, proximal soothing behaviors, and distress-promoting behaviors as predictors, and infant pain behaviors during the reactivity phase as the outcome. Separate regressions were conducted for the two-month, six-month, and 12-month vaccination data. All assumptions for the multivariate analyses were met. A summary of the multiple regression analyses for infant pain reactivity is provided in Table 3.

#### 3.1.1. Infant Pain Behavior during the Reactivity Phase at Two Months

During two-month vaccinations, parent behaviors accounted for 11.5% of the variance in infant pain during the reactivity phase (adjusted R^2^ = 0.12, F(3,63) = 3.87, *p* = 0.01). Parent distress-promoting behaviors were the only significant predictor of pain behavior (β = 0.33, *p* = 0.01), uniquely accounting for 10.2% of the variance in pain behaviors during this phase (*sr*^2^ = 0.10). This result suggests that higher levels of distress-promoting behaviors are associated with greater infant pain during the reactivity phase.

#### 3.1.2. Infant Pain Behavior during the Reactivity Phase at Six Months

During six-month vaccinations, parent behaviors accounted for 27.6% of the variance in infant pain during the reactivity phase (adjusted R^2^ = 0.28, F(3,70) = 10.26, *p* < 0.001). Parent proximal soothing (β = 0.23, *p* = 0.02) and distress-promoting behaviors (β = 0.54, *p* < 0.001) both predicted pain behavior, with more proximal soothing behaviors and distress-promoting behaviors both being associated with greater infant pain during this phase. Squared semi-partial correlations revealed that distress-promoting behaviors uniquely accounted for 24.6% of the variance in pain during the reactivity phase, whereas proximal soothing accounted for 5.4% of the variance.

#### 3.1.3. Infant Pain Behavior during the Reactivity Phase at 12 Months

During 12-month vaccinations, parent behaviors accounted for 12.9% of the variance in infant pain during the reactivity phase (adjusted R^2^ = 0.13, F(3,75) = 4.84, *p* = 0.004). Both parent emotional availability (β = −0.25, *p* = 0.04) and distress-promoting behaviors (β = 0.25, *p* = 0.03) predicted pain behaviors, with greater emotional availability predicting lower pain, and more distress-promoting behaviors predicting higher pain during the reactivity phase. Emotional availability uniquely accounted for 5.2% of the variance in pain behavior (*sr*^2^ = 0.05), while distress-promoting behavior uniquely accounted for 5.3% of the variance (*sr*^2^ = 0.05).

### 3.2. Research Question 2: Predicting Infant Pain Behavior during the Regulation Phase

To investigate the relationship between sensitive and insensitive parent variables, and infant pain behavior during the regulation phase, multiple linear regressions were conducted using parent emotional availability, proximal soothing behaviors, and distress-promoting behaviors as predictors, and infant pain behaviors during the regulation phase as the outcome. Separate regressions were conducted for the two-month, six-month, and 12-month vaccination data. All assumptions for the multivariate analyses were met. A summary of the multiple regression analyses for infant pain regulation is provided in Table 4.

#### 3.2.1. Infant Pain Behavior during the Regulation Phase at Two Months

During two-month vaccinations, parent behaviors accounted for 26.6% of the variance in infant pain behaviors during the regulation phase (adjusted R^2^ = 0.27, F(3,63) = 8.99, *p* < 0.001). Parent distress-promoting behaviors were the only significant predictor of infant pain regulation (β = 0.46, *p* < 0.001), uniquely accounting for 20.4% of the variance in pain behaviors during this phase (*sr*^2^ = 0.20). This result suggests that higher levels of distress-promoting behaviors are associated with greater pain during the regulation phase.

#### 3.2.2. Infant Pain Behavior during the Regulation Phase at Six Months.

During six-month vaccinations, parent behaviors accounted for 41.6% of the variance in infant pain behaviors during the regulation phase (adjusted R^2^ = 0.42, F(3,70) = 18.31, *p* < 0.001). Parent proximal soothing (β = 0.31, *p* = 0.001) and distress-promoting behaviors (β = 0.64, *p* < 0.001) both predicted infant pain behaviors during the regulation phase, with more proximal soothing behaviors and distress-promoting behaviors both being associated with greater infant pain. Distress-promoting behaviors uniquely accounted for 34.8% of the variance in pain behaviors during the regulation phase (*sr*^2^ = 0.35), while proximal soothing accounted for 9.4% of the variance (*sr*^2^ = 0.09).

#### 3.2.3. Infant pain behavior during the regulation phase at 12 months.

During 12-month vaccinations, parent behaviors accounted for 26% of the variance in infant pain behaviors during the regulation phase (adjusted R^2^ = 0.26, F(3,75) = 10.13, *p* < 0.001). Paralleling the six-month results, parent proximal soothing (β = 0.31, *p* = 0.003) and distress-promoting behaviors (β = 0.48, *p* < 0.001) both predicted infant pain behavior during the regulation phase, with more proximal soothing behaviors and distress-promoting behaviors both being associated with greater pain. Distress-promoting behaviors uniquely accounted for 20.3% of the variance in pain behaviors during the regulation phase (*sr*^2^ = 0.20), while proximal soothing accounted for 9.1% of the variance (*sr*^2^ = 0.09).

## 4. Discussion

The goal of the current study was to investigate the relative contributions of potentially sensitive and insensitive parent behaviors to the prediction of infant pain-related behavioral distress reactivity and regulation during vaccinations occurring during the first year of life. Previous work from the OUCH cohort identified strong relationships between parent distress-promoting behaviors and infant pain-related distress during the regulation phase. Moreover, smaller relationships were also seen between both proximal soothing and emotional availability and infant pain-related distress during the regulation phase. However, the relative contributions of each of these variables in the prediction of infant pain outcomes were not examined to date. Identifying the aspects of parent behavior that have the strongest associations with pain outcomes is important for informing pain management strategies within the routine vaccination context, particularly given the importance of the first year of life for laying the foundation for infant distress regulation skills.

In line with our first hypothesis, parent behaviors consistently accounted for more overall variance in infant pain behaviors during the regulation phase, when compared with infant pain behaviors during the reactivity phase. The magnitude of this difference was large. Parent behaviors accounted for twice the amount of variance in regulation-phase pain, when compared with reactivity-phase pain, at two of the three time points (two months and 12 months). This finding lends support to theories purporting that pain reactivity is driven more by biological factors and previous experiences, as opposed to concurrent parent behaviors, and reinforces the need to differentiate between these two distinct phases of infants’ pain experience when studying pain behavior in this context [6,7].

When entered as a predictor alongside parent emotional availability and proximal soothing behaviors, parent distress-promoting behaviors were consistently the strongest predictor of infant pain during both the reactivity and regulation phases. Differences in the relative contribution of distress-promoting behaviors, when compared with more “optimal” aspects of parent behavior, were substantial. During the regulation phase in particular, the contributions of proximal soothing and emotional availability were very small (<10%) once the effect of distress-promoting behaviors was accounted for. Specifically, distress-promoting behaviors accounted for at least double (and up to 4×) the variance accounted for by proximal soothing behaviors, and at least six times (and up to 100×) the variance accounted for by emotional availability across the three time points. This finding supports our second hypothesis, and makes a novel contribution to our understanding of the mechanisms through which parent behaviors influence infant acute pain outcomes. It also replicates findings from previous studies that identified stronger relationships between parent distress-promoting behaviors (when compared with coping-promoting behaviors) and pain-related distress in in preschoolers [19] and older children [20,21]. Taken together, these findings suggest that teaching parents to avoid engaging in suboptimal behaviors may be more impactful than solely emphasizing optimal behaviors in the management of vaccination pain. This finding has important implications for the way that infant vaccination pain is studied and managed. We showed that suboptimal parent behaviors, as measured by a checklist of eight observable parent behaviors, were the most robust predictor of infant pain outcomes. Given that many commonly used measures of parent pain management behaviors require substantial time commitments (e.g., training and micro-coding) and/or clinical expertise to be used appropriately, the use of this new checklist of insensitive parent behaviors may offer a more feasible and beneficial way for both researchers and healthcare professionals to study and measure parent pain management behaviors.

We also observed some interesting developmental trends in our results. Firstly, the regression model for six-month vaccination pain accounted for a greater amount of total variance in behaviors during the regulation phase (adjusted R^2^ = 42%) when compared with the models for two-month (27%) and 12-month (26%) vaccinations. An identical trend was seen in the models predicting behavior during the reactivity phase. This was unexpected, given that attachment theory would predict that parent behavior (e.g., emotional availability) would have the strongest relationships with pain outcomes toward the end of the first year of life [13,14]. Of note, mean infant pain-behavior scores were lower during the regulation phase at six months, when compared with those at two months and 12 months, even though the mean number of needles received was similar across visits. Despite this difference, the trends within the model remained consistent with other ages, and indicated that distress-promoting behaviors were still the strongest predictor within the model, while emotional availability was the weakest predictor. Secondly, we found that, while proximal soothing behaviors (i.e., rocking and physical comfort) became stronger predictors of regulation-phase pain toward the end of the first year (9%) when compared with the beginning (4%), this relationship was consistently positive, indicating that more proximal soothing was associated with greater pain. Although consistent with previous research on the OUCH cohort [10,12], finding a positive relationship between these two variables is inconsistent, with a previous study on a different infant sample which found significant negative relationships between proximal soothing and infant regulation-phase pain [6]. Our finding may be explained by the fact that parents who had more reactive infants (i.e., higher distress during reactivity phase) could be expected to use more proximal soothing behaviors during the regulation phase. 

Finally, we found that emotional availability made negligible contributions to pain outcomes when other aspects of parent behaviors were put in the model. While previous work with the OUCH cohort identified relationships between emotional availability and pain outcomes, these studies did not examine the concurrent contributions of emotional availability in the context of other parent factors [14,15,19]. Given that many of the behaviors accounted for by our measure of distress-promoting behaviors are reflective of a low degree of emotional attunement between parent and infant, and can, therefore, be conceptualized as being indicative of parent insensitivity, our finding is not surprising. In our study, parent emotional availability ratings were based on observation of the entire vaccination appointment (pre- and post-needle), whereas distress-promoting behaviors were based on parent behaviors specifically in the context of moderate-to-high infant distress post-needle. Thus, our measure of distress-promoting behaviors may be a more specific measure of parent emotional availability in the context of infant pain-related distress.

Our results should be interpreted within the context of some limitations. Firstly, our sample consisted primarily of university-educated, two-parent families, which limits the generalizability of our findings. Secondly, it is possible that parents’ behaviors were affected by nature of being videotaped during the vaccination appointments. Thirdly, due to the nature of the measures used, parent behavior variables used in the models predicting infant pain reactivity were based on observations of parent behavior that occurred beyond the reactivity period. However, given that our analyses aimed to identify the relative contribution of each parent behavior in relation to other behaviors, this limitation is not expected to have impacted our conclusions. Finally, while emotional availability and proximal soothing are both referred to as sensitive parent behaviors, it should be noted that proximal soothing is considered a sensitive behavior only in the context of the infant welcoming that soothing. There are instances where too much rocking and physical comforting may not be sensitive. This limitation is not the case for the other two parent behavioral measures which are designed to focus on infant needs, and are, therefore, clearly sensitive or insensitive.

In conclusion, the results of this study showed that parent distress-promoting behaviors in an infant vaccination context have the strongest relationships with infant pain reactivity and regulation, in relation to other previously investigated aspects of parent behavior. Our study provides support for the use of a brief checklist of suboptimal parent behaviors to effectively capture the influence of parents on infant pain outcomes. From a broader clinical perspective, our results suggest that teaching parents what not to do may be at least as important as telling them what to do. Based on our findings, educating parents on the impact of distress-promoting behaviors is recommended to supplement parent education on effective pain management strategies during infant vaccinations (e.g., breastfeeding [26] and non-nutritive sucking [27,28]). Future directions for this line of research should include efforts to train primary care health professionals to screen for these suboptimal parent behaviors, with the goal of supporting parents in effectively soothing their infants’ pain post-vaccination.

## Figures and Tables

**Table 1:** Demographic characteristics.

Characteristic	2 Months	6 Months	12 Months
	(*n* = 67) %	(*n* = 74) %	(*n* = 79) %
Infant gender (% male)	56.7	56.8	54.4
Parent(s) present at vaccination			
Mother	40.3	63.5	59.5
Mother and father	41.8	27.0	30.4
Father	0	1.4	2.5
Parent(s) and grandparent(s)	13.4	5.4	5.1
Parent(s) and other	4.5	2.7	2.6

Note: Mothers did the majority of caregiving in 91–93% of cases across appointments.

**Table 2:** Descriptive statistics.

Variable	2 Months	6 Months	12 Months
	(*n* = 67)	(*n* = 74)	(*n* = 79)
	Mean (SD)		
Infant pain behaviors—reactivity phase ^a^	8.72 (1.00)	8.36 (1.43)	8.57 (0.80)
Infant pain behaviors—regulation phase ^b^	12.13 (4.60)	8.89 (4.54)	11.90 (4.06)
Parent proximal soothing ^c^	0.59 (0.47)	0.41 (0.37)	0.40 (0.31)
Parent emotional availability ^d^	90.45 (12.13)	92.85 (11.36)	90.16 (11.51)
Parent distress-promoting behavior ^e^	1.84 (1.30)	1.59 (1.19)	2.24 (1.16)

^a^ range 0–10; ^b^ range 0–20; ^c^ range 0–2; ^d^ range 28–116; and ^e^ range 0–8.

**Table 3:** Summary of multiple regression analysis for infant pain behavior during the reactivity phase.

Variable	*B*	SE	β	*p*	*sr* ^2^
Infant pain behavior (reactivity phase)—2 months (Adjusted R^2^ = 0.12)					
Parent proximal soothing	0.22	0.26	0.10	0.40	0.01
Parent emotional availability	−0.01	0.01	−0.16	0.21	0.02
Parent distress-promoting behavior	0.25	0.09	0.33	0.01	0.10
Infant pain behavior (reactivity phase)—6 months (Adjusted R^2^ = 0.28)					
Parent proximal soothing	0.89	0.38	0.23	0.02	0.05
Parent emotional availability	0.01	0.01	0.07	0.54	0.00
Parent distress-promoting behavior	0.65	0.13	0.54	<0.001	0.25
Infant pain behavior (reactivity phase)—12 months (Adjusted R^2^ = 0.13)					
Parent proximal soothing	0.28	0.28	0.11	0.31	0.01
Parent emotional availability	−0.02	0.01	−0.25	0.04	0.05
Parent distress-promoting behavior	0.17	0.08	0.25	0.03	0.05

*B* = unstandardized regression coefficient; SE = standard error of unstandardized regression coefficient; β = standardized regression coefficient; and *sr*^2^ = squared semi-partial correlation coefficient.

**Table 4:** Summary of multiple regression analysis for infant pain behavior during the regulation phase.

Variable	*B*	*SE*	β	*p*	*sr* ^2^
Infant pain behavior (regulation phase)—2 months (Adjusted R^2^ = 0.27)					
Parent proximal soothing	2.04	1.10	0.21	0.07	0.04
Parent emotional availability	0.07	0.04	0.17	0.13	0.03
Parent distress-promoting behavior	1.64	0.38	0.46	<0.001	0.20
Infant pain behavior (regulation phase)—6 months (Adjusted R^2^ = 0.42)					
Parent proximal soothing	3.75	1.09	0.31	0.001	0.09
Parent emotional availability	0.05	0.04	0.11	0.25	0.01
Parent distress-promoting behavior	2.44	0.37	0.64	<0.001	0.35
Infant pain behavior (regulation phase)—12 months (Adjusted R^2^ = 0.26)					
Parent proximal soothing	4.05	1.31	0.31	0.003	0.09
Parent emotional availability	0.02	0.04	0.05	0.65	0.00
Parent distress-promoting behavior	1.69	0.37	0.48	<0.001	0.20

*B* = unstandardized regression coefficient; SE = standard error of unstandardized regression coefficient; β = standardized regression coefficient; and *sr*^2^ = squared semi-partial correlation coefficient.
